# Proving the Safety of Highly-Available Distributed Objects

**DOI:** 10.1007/978-3-030-44914-8_20

**Published:** 2020-04-18

**Authors:** Sreeja S. Nair, Gustavo Petri, Marc Shapiro

**Affiliations:** ETH Zurich, Zurich, Switzerland; 9 Sorbonne Université—LIP6 & Inria, Paris, France; 10 ARM Research, Cambridge, UK

**Keywords:** Replicated objects, Consistency, Automatic verification, Distributed application design, Tool support

## Abstract

To provide high availability in distributed systems, object replicas allow concurrent updates. Although replicas eventually converge, they may diverge temporarily, for instance when the network fails. This makes it difficult for the developer to reason about the object’s properties, and in particular, to prove invariants over its state. For the subclass of state-based distributed systems, we propose a proof methodology for establishing that a given object maintains a given invariant, taking into account any concurrency control. Our approach allows reasoning about individual operations separately. We demonstrate that our rules are sound, and we illustrate their use with some representative examples. We automate the rule using Boogie, an SMT-based tool.

## 1 Introduction

Many modern applications serve users accessing shared data in different geographical regions. Examples include social networks, multi-user games, cooperative engineering, collaborative editors, source-control repositories, or distributed file systems. One approach would be to store the application's data (which we call object) in a single central location, accessed remotely. However, users far from the central location would suffer long delays and outages.

Instead, the object is *replicated* to several locations. A user accesses the closest available replica. To ensure *availability*, an update must not synchronise across replicas; otherwise, when a network partition occurs, the system would block. Thus, a replica executes both queries and updates locally, and propagates its updates to other replicas asynchronously.

Updates at different locations are concurrent; this may cause replicas to diverge, at least temporarily. Replicas may diverge, but if the system ensures Strong Eventual Consistency (SEC), this ensures that replicas that have received the same set of updates have the same state [25], simplifying the reasoning.

The replicated object may also require to maintain some (application-specific) *invariant*, an assertion about the object. We say a state is safe if the invariant is true in that state; the system is safe if every reachable state is safe. In a sequential system, this is straightforward (in principle): if the initial state is safe, and the final state of every update individually is safe, then the system is safe. However, these conditions are not sufficient in the replicated case, because concurrent updates at different replicas may interfere with one another. This can be fixed by synchronising between some or all types of updates. To maximise availability and latency, such synchronisation should be minimised. In this paper, we propose a proof methodology to ensure that a given object is system-safe, for a given invariant and a given amount of concurrency control. In contrast to previous works, we consider state-based objects.[1] Indeed, the specific properties of state-based propagation enable simple modular reasoning despite concurrency, thanks to the concept of *concurrency invariant*. Our proof methodology derives the concurrency invariant automatically from the sequential specification. Now, if the initial state is safe, and every update maintains both the application invariant and the concurrency invariant, then every reachable state is safe, even in concurrent executions, regardless of network partitions. We have developed a tool named Soteria, to automate our proof methodology. Soteria analyses the specification to detect concurrency bugs and provides counterexamples.

The contributions of this paper are as follows:

- We propose a novel proof system specialised to proving the safety of available objects that converge by propagating state. This specialisation supports modular reasoning, and thus it enables automation.
- We demonstrate that this proof system is sound. Moreover, we provide a simple semantics for state-propagating systems that allows us to ignore network messages altogether.
- We present Soteria, to the best of our knowledge the first tool supporting the verification of program invariants for state-based replicated objects. When Soteria succeeds it ensures that every execution, whether replicas are partitioned or concurrent, is safe.
- We present a number of representative case studies, which we run through Soteria.

## 2 Background

As a running example, consider a simple auction system (for simplicity, we consider a single auction). An auction object is composed of the following parts:

- Its `Status`, that can move from initial state `INVALID` (under preparation) to `ACTIVE` (can receive bids) and then to `CLOSED` (no more bids accepted).
- The `Winner` of the auction, that is initially $\perp$ and can become the bid taking the highest amount. In case of ties, the bid with the lowest id wins.
- The set of `Bids` placed, that is initially empty. A bid is a tuple composed of
  - `BidId`: A unique identifier
  - `Placed`: A boolean flag to indicate whether the bid has been placed or not. Initially, it is `FALSE`. Once placed, a bid cannot be withdrawn.
  - The monetary `Amount` of the bid; this cannot be modified once the bid is created.

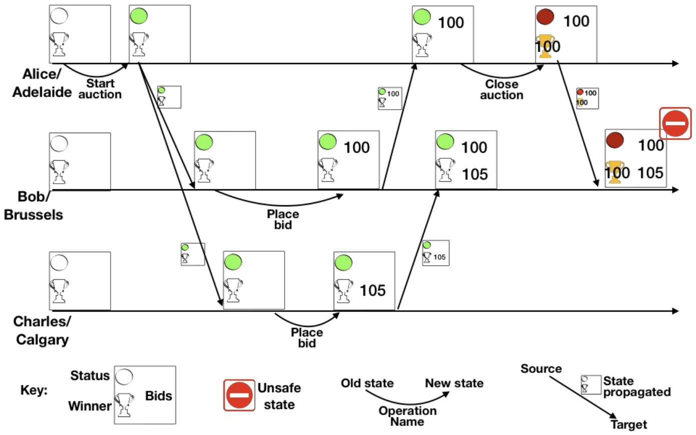

Fig. 1: Evolution of state of an auction object

Figure 1 illustrates how the auction state evolves over time. The state of the object is geo-replicated at data centers in Adelaide, Brussels, and Calgary. Users at different locations can start an auction, place bids, close the auction, declare a winner, inspect the local replica, and observe if a winner is declared and who it is. The updates are propagated asynchronously to other replicas. All replicas will eventually agree on the same auction status, the same set of bids and the same winner.

There are two basic approaches to propagating updates. The operation-based approach applies an update to some origin replica, then transmits the operation itself to be replayed at other replicas. If messages are delivered in causal order, exactly once, and concurrent operations are commutative, then two replicas that received the same updates reach the same state (this is the Strong Eventual Consistency guarantee, or SEC) [25].

The state-based approach applies an update to some origin replica. Occasionally, a replica sends its full state to some other replica, which *merge*s the received state into its own. If the state space forms a monotonic semi-lattice, an update is an inflation (its output state is not lesser than the input state), and *merge* computes the least-upper-bound of the local and received states, then SEC is guaranteed [25]. As long as every update eventually reaches every replica, messages may be dropped, re-ordered or duplicated, and the set of replicas may be unknown. Due to these relaxed requirements, state-based propagation is widely used in industry. Figure 1 shows the state-based approach with local operations and merges. Alternatives exist where only a delta of the state —that is, the portion of the state not known to be part of the other replicas— is sent as a message [1]; since this is an optimisation, it is of no consequence to the results of this paper.

[1] As opposed to operation-based. These terms are defined in Section 2.

Looking back to Figure 1, we can see that replicas diverge temporarily. This temporary divergence can lead to an unsafe state, in this case declaring a wrong winner. This correctness problem has been addressed before; however, previous works mostly consider the operation-based propagation approach [11, 13, 19, 24].

## 3 System Model

In this section, we first introduce the object components, explain the underlying system model informally, and then formalise the operational semantics.

### 3.1 General Principles

An object consists of a state, a set of operations, a merge function and an invariant. Figure 1 illustrates three replicas of an auction object, at three different locations, represented by the horizontal lines. The object evolves through a set of states. Each line depicts the evolution of the state of the corresponding replica; time flows from left to right.

*State.* A distributed system consists of a number of servers, with disjoint memory and processing capabilities. The servers might be distributed over geographical regions. A set of servers at a single location stores the state of the object. This is called a single *replica*. The object is replicated at different geographical locations, each location having a full copy of the state. In the simplest case (for instance at initialisation) the state at all replicas will be identical. The state of each replica is called a *local state*. The global view, comprising all local states is called the *global state*.

*Operations.* Each replica may perform the operations defined for the object. To support availability, an operation modifies the local state at some arbitrary replica, the *origin replica* for that operation, without synchronising with other replicas (the cost of synchronisation being significant at scale). An operation might consist of several changes; these are applied to the replica as a single atomic unit.

Executing an operation on its origin replica has an immediate effect. However, the state of the other replicas, called *remote replicas*, remains unaltered at this point. The remote replicas get updated when the state is eventually propagated. An immediate consequence of this execution model is that in the presence of concurrent operations, replicas can reach different states, i.e. they diverge.

Let us illustrate this with our example in Figure 1. Initially, the auction is yet to start, the winner is not declared and no bids are placed. By default, a replica can execute any operation - `start_auction`, `place_bid`, and `close_auction` - locally without synchronising with other replicas. We see that the local states of replicas occasionally diverge. For example at the point where operation `close_auction` completes at the Adelaide replica, the Adelaide replica is aware of only a $100 bid, the Brussels replica has two bids, and the Calgary replica observes only one bid for $105.

*State Propagation.* A replica occasionally propagates its state to other replicas in the system and a replica receiving a remote state *merges* it into its own.

In Figure 1, the arrows crossing between replicas represent the delivery of a message containing the state of the source replica, to be merged into the target replica. A message is labelled with the state propagated. For instance, the first message delivery at the Brussels replica represents the result of updating the local state (setting auction status to `ACTIVE`), with the state originating in the replica at Adelaide (auction started).

Similar to the operations, a merge is atomic. In Figure 1, Alice closes the auction at the Adelaide replica. This atomically sets the status of the auction to `CLOSED` and declares a winner from the set of bids it is aware of. The updated auction state and winner are transmitted together. Merging is performed atomically by the Brussels replica.[2]

We now specify the `merge` operation for an auction. The receiving replica's local state is denoted $\sigma$ = (status, winner, Bids), the received state is denoted $\sigma'$ = (status′, winner′, Bids′) and the result of merge is denoted as $\sigma_{new}$ = ($\text{status}_{new}$, $\text{winner}_{new}$, $\text{Bids}_{new}$).

```
merge((status,winner,Bids),(status′,winner′,Bids′)) :
  status_new  := max(status,status′)
  winner_new  := winner′ ≠ ⊥ ? winner′ : winner
  for (b in Bids ∪ Bids′)
    Bids_new.b.placed  := Bids.b.placed ∨ Bids′.b.placed
    Bids_new.b.amount  := max(Bids.b.amount, Bids′.b.amount)
```

Furthermore, we require the operations and merge to be defined in a way that ensures convergence. We discuss the relevant properties later in Section 6.1.

*Invariants.* An invariant is an assertion that must evaluate to true in every local state of every replica. Although evaluated locally at each replica, the invariant is in effect global, since it must be true at all replicas, and replicas eventually converge. For our running example, the invariant can be stated as follows:

- Only an active auction can receive bids, and
- the highest unique bid wins when the auction closes (breaking ties using bid identifiers).

This condition must hold true in all possible executions of the object.

### 3.2 Notations and Assumptions

First, we introduce some notations and assumptions:

- We assume a fixed set of replicas, ranged over with the meta-variable $\mathtt{r} \in \mathtt{R}$ sampled from the domain of unique replica names $\mathtt{R}$.
- We denote a local state with the meta-variable $\sigma \in \Sigma$ ranged over the domain of states of the object $\Sigma$.

[2] We see that this leads to an unsafe state, we discuss this in detail in Section 4.2

- The *local semantic* function $[\![]\!]$ takes an operation and a state, and returns the state after applying the operation. We write $[\![\texttt{op}]\!](\sigma) = \sigma_{new}$ for executing operation op on state $\sigma$ resulting in a new state $\sigma_{new}$.
- $\Omega$ denotes a partial function returning the current state of a replica. For instance $\Omega(\texttt{r}) = \sigma$ means that in global state $\Omega$, replica r is in local state $\sigma$. We will use the notation $\Omega[\texttt{r} \leftarrow \sigma]$ to denote the global state resulting from replacing the local state of replica r with $\sigma$. The local state of all other replicas remains unchanged in the resulting global state.[3]
- A message propagating states between replicas is denoted $\langle\ \texttt{r} \xrightarrow{\sigma} \texttt{r}'\ \rangle$. This represents the fact that replica r has sent a message (possibly not yet received) to replica $\texttt{r}'$, with the state $\sigma$ as its payload. The meta-variable M denotes the messages in transit in the network.
- In the following sub-section, we will utilise a set of states to record the history of the execution. The set of past states will be ranged over with the variable $\texttt{S} \in \mathbb{P}(\Sigma)$.
- All replicas are assumed to start in the same initial state $\sigma_i$. Formally, for each replica $\texttt{r} \in \mathsf{dom}(\Omega_i)$ we have $\Omega_i(\texttt{r}) = \sigma_i$.

### 3.3 Operational Semantics

In this and the following subsections we will present two semantics for systems propagating states. Importantly, while the first semantics takes into account the effects of the network on the propagation of the states, and is hence an accurate representation of the execution of systems with state propagation, we will show in the next subsection that reasoning about the network is unnecessary in this kind of system. We will demonstrate this claim by presenting a much simpler semantics in which the network is abstracted away. The importance of this reduction is that the number of events to be considered, both when conducting proofs and when reasoning about applications, is greatly reduced. As informal evidence of this claim, we point at the difference in complexity between the semantic rules presented in Figure 2 and Figure 3. We postpone the equivalence argument to Theorem 1.

Figure 2 presents the semantic rules describing what we shall call the *precise semantics* (we will later present a more abstract version) defining the transition relations describing how the state of the object evolves.

The figure defines a semantic judgement of the form $(\Omega, \texttt{M}) \rightarrow (\Omega_{new}, \texttt{M}_{new})$ where $(\Omega, \texttt{M})$ is a configuration where the replica states are given by $\Omega$ as shown above, and M is a set of messages that have been transmitted by different replicas and are pending to be received by their target replicas.

Rule Operation presents the state transition resulting from a replica r executing an operation op. The operation queries the state of replica r, evaluates the semantic function for operation op and updates its state with the result. The

[3] This notation of a global state is used only to explain and prove our proof rule. In fact, the rule is based only on the local state of each replica.

$$\textsc{Operation}\quad \frac{\Omega(\mathtt{r}) = \sigma \qquad [\![\mathsf{op}]\!](\sigma) = \sigma_{new} \qquad \Omega_{new} = \Omega[\mathtt{r} \leftarrow \sigma_{new}]}{(\Omega, \mathtt{M}) \to (\Omega_{new}, \mathtt{M})}$$

$$\textsc{Send}\quad \frac{\Omega(\mathtt{r}) = \sigma \qquad \mathtt{r}' \in \mathsf{dom}(\Omega) \setminus \{\mathtt{r}\} \qquad \mathtt{M}_{new} = \mathtt{M} \cup \{\langle\, \mathtt{r} \xrightarrow{\sigma} \mathtt{r}' \,\rangle\}}{(\Omega, \mathtt{M}) \to (\Omega, \mathtt{M}_{new})}$$

$$\textsc{Merge}\quad \frac{\begin{array}{c}\Omega(\mathtt{r}) = \sigma \\ \mathtt{M}_{new} = \mathtt{M} \setminus \{\langle\, \mathtt{r}' \xrightarrow{\sigma'} \mathtt{r} \,\rangle\} \qquad [\![\mathtt{merge}]\!](\sigma, \sigma') = \sigma_{new} \qquad \Omega_{new} = \Omega[\mathtt{r} \leftarrow \sigma_{new}]\end{array}}{(\Omega, \mathtt{M}) \to (\Omega_{new}, \mathtt{M}_{new})}$$

$$\textsc{Op \& Broadcast}\quad \frac{\begin{array}{c}\Omega(\mathtt{r}) = \sigma \qquad [\![\mathsf{op}]\!](\sigma) = \sigma_{new} \qquad \Omega_{new} = \Omega[\mathtt{r} \leftarrow \sigma_{new}] \\ \mathtt{M}_{new} = \mathtt{M} \cup \{\ \langle\, \mathtt{r} \xrightarrow{\sigma_{new}} \mathtt{r}' \,\rangle \mid \mathtt{r}' \in \mathsf{dom}(\Omega) \setminus \{\mathtt{r}\}\ \}\end{array}}{(\Omega, \mathtt{M}) \to (\Omega_{new}, \mathtt{M}_{new})}$$

$$\textsc{Merge \& Broadcast}\quad \frac{\begin{array}{c}\Omega(\mathtt{r}) = \sigma \\ \mathtt{M}_{new} = \mathtt{M} \setminus \{\langle\, \mathtt{r}' \xrightarrow{\sigma'} \mathtt{r} \,\rangle\} \qquad [\![\mathtt{merge}]\!](\sigma, \sigma') = \sigma_{new} \qquad \Omega_{new} = \Omega[\mathtt{r} \leftarrow \sigma_{new}] \\ \mathtt{M}_{new'} = \mathtt{M}_{new} \cup \{\ \langle\, \mathtt{r} \xrightarrow{\sigma_{new}} \mathtt{r}'' \,\rangle \mid \mathtt{r}'' \in \mathsf{dom}(\Omega) \setminus \{\mathtt{r}\}\ \}\end{array}}{(\Omega, \mathtt{M}) \to (\Omega_{new}, \mathtt{M}_{new'})}$$

Fig. 2: Precise Operational Semantics: Messages set of messages $\mathtt{M}$ does not change. The second rule, Send, represents the non-deterministic sending of the state of replica $\mathtt{r}$ to replica $\mathtt{r}'$. The rule has no other effect than to add a message to the set of pending messages $\mathtt{M}$. The Merge rule picks any message, $\langle\, \mathtt{r}' \xrightarrow{\sigma'} \mathtt{r} \,\rangle$, in the set of pending messages $\mathtt{M}$, and applies the merge function to the destination replica with the state in the payload of the message, removing $\langle\, \mathtt{r}' \xrightarrow{\sigma'} \mathtt{r} \,\rangle$ from $\mathtt{M}$.

The final two rules, Op & Broadcast and Merge & Broadcast represent the specific case when the states are immediately sent to all replicas. These rules are not strictly necessary since they are subsumed by the application of either Operation or Merge followed by one Send per replica. We will, however, use them to simplify a simulation argument in what follows.

We remark at this point that no assumptions are made about the duplication of messages or the order in which messages are delivered. This is in contrast to other works on the verification of properties of replicated objects [11, 13]. The reason why this assumption is not a problem in our case is that the least-upper-bound assumption of the `merge` function, as well as the inflation assumptions on the states considered in Item 2 (Section 6.1) mean that delayed messages have no effect when they are merged.

$$\textsc{Operation}\quad \frac{\Omega(\mathtt{r}) = \sigma \qquad [\![\mathtt{op}]\!](\sigma) = \sigma_{new} \qquad \Omega_{new} = \Omega[\mathtt{r} \leftarrow \sigma_{new}]}{(\Omega, \mathtt{S}) \rightarrow (\Omega_{new}, \mathtt{S} \cup \{\sigma_{new}\})}$$

$$\textsc{Merge}\quad \frac{\Omega(\mathtt{r}) = \sigma \qquad \sigma' \in \mathtt{S} \qquad [\![\mathtt{merge}]\!](\sigma, \sigma') = \sigma_{new} \qquad \Omega_{new} = \Omega[\mathtt{r} \leftarrow \sigma_{new}]}{(\Omega, \sigma) \rightarrow (\Omega_{new}, \mathtt{S} \cup \{\sigma_{new}\})}$$

Fig. 3: Semantic Rules with a History of States

As customary we will denote with $(\Omega, \mathtt{M}) \xrightarrow{*} (\Omega_{new}, \mathtt{M}_{new})$ the repeated application of the semantic rules zero or more times, from the state $(\Omega, \mathtt{M})$ resulting in the state $(\Omega_{new}, \mathtt{M}_{new})$.

It is easy to see how the example in Figure 1 proceeds according to these rules for the auction.

The following lemma,[4] to be used later, establishes that whenever we use only the broadcast rules, for any intermediate state in the execution, and for any replica, when considering the final state of the trace, either the replica has already observed a fresher version of the state in the execution, or there is a message pending for it with that state. This is an obvious consequence of broadcasting.

**Lemma 1.** *If we consider a restriction to the semantics of Figure 2 where instead of applying the* Operation *rule of Figure 2 we apply the* Op & Broadcast *rule always, and instead of applying the* Merge *rule we apply* Merge & Broadcast *always, we can conclude that given an execution starting from an initial global state $\Omega_i$ with*

$$(\Omega_i, \emptyset) \xrightarrow{*} (\Omega, \mathtt{M}) \xrightarrow{*} (\Omega_{new}, \mathtt{M}_{new})$$

*for any two replicas $\mathtt{r}$ and $\mathtt{r}'$ and a state $\sigma$ such that $\Omega(\mathtt{r}) = \sigma$, then either:*

- $\Omega_{new}(\mathtt{r}') \geq \sigma$, *or*
- $\langle\ \mathtt{r} \xrightarrow{\sigma} \mathtt{r}'\ \rangle \in \mathtt{M}_{new}$.

### 3.4 Operational Semantics with State History

We now turn our attention to a simpler semantics where we omit messages from configurations, but instead, we record in a separate set all the states occurring in any replica throughout the execution.

The semantics in Figure 3 presents a judgement of the form $(\Omega, \mathtt{S}) \rightarrow (\Omega_{new}, \mathtt{S}_{new})$ between configurations of the form $(\Omega, \mathtt{S})$ as before, but where the set of messages is replaced by a set of states denoted with the meta-variable $\mathtt{S} \in \mathbb{P}(\Sigma)$.

[4] The proofs for the lemmas are included in the extended version[23].

The rules are simple. Operation executes an operation as before, and it adds the resulting new state to the set of observed states. The rule Merge non-deterministically selects a state in the set of states and it merges a non-deterministically chosen replica with it. The resulting state is also added to the set of observed states.

**Lemma 2.** *Consider a state* $(\Omega, \mathtt{S})$ *reachable from an initial global state* $\Omega_i$ *with the semantics of Figure 3. Formally:* $(\Omega_i, \{\sigma_i\}) \xrightarrow{*} (\Omega, \mathtt{S})$. *We can conclude that the set of recorded states in the final configuration* $\mathtt{S}$ *includes all of the states present in any of the replicas*

$$\Big( \bigcup_{\mathtt{r} \in \mathsf{dom}(\Omega)} \{\Omega(\mathtt{r})\} \Big) \subseteq \mathtt{S}$$

### 3.5 Correspondence between the semantics

In this section, we show that removing the messages from the semantics, and choosing to record states instead renders the same executions. To that end, we will define the following relation between configurations of the two semantics which will be later shown to be a bisimulation.

**Definition 1 (Bisimulation Relation).** *We define the relation* $\mathcal{R}_{\Omega_i}$ *between a configuration* $(\Omega, \mathtt{M})$ *of the semantics of Figure 2 and a configuration* $(\Omega, \mathtt{S})$ *of the semantics of Figure 3 parameterized by an initial global state* $\Omega_i$ *and denoted by*

$$(\Omega, \mathtt{M})\ \mathcal{R}_{\Omega_i}\ (\Omega, \mathtt{S})$$

*when the following conditions are met:*

1. $(\Omega_i, \emptyset) \xrightarrow{*} (\Omega, \mathtt{M})$, *and*
2. $(\Omega_i, \{\sigma_i\}) \xrightarrow{*} (\Omega, \mathtt{S})$, *and*
3. $\{\ \sigma \mid \langle\ \mathtt{r} \xrightarrow{\sigma} \mathtt{r}'\ \rangle \in \mathtt{M}\ \} \subseteq \mathtt{S}$

In other words, two states represented in the two configurations are related if both are reachable from an initial global state and all the states transmitted by the messages ($\mathtt{M}$) is present in the history ($\mathtt{S}$).

We can now show that this relation is indeed a bisimulation. We first show that the semantics of Figure 3 simulates that of Figure 2. That is, all behaviours produced by the precise semantics with messages can also be produced by the semantics with history states. This is illustrated in the commutative diagram of Figure 4a and Figure 4b, where the dashed arrows represent existentially quantified components that are proven to exist in the theorem.

**Lemma 3 (State-semantics simulates Messages-semantics).** *Consider a reachable state* $(\Omega, \mathtt{M})$ *from the initial state* $\Omega_i$ *in the semantics of Figure 2. Consider moreover that according to that semantics there exists a transition of the form*

$$(\Omega, \mathtt{M}) \rightarrow (\Omega_{new}, \mathtt{M}_{new})$$

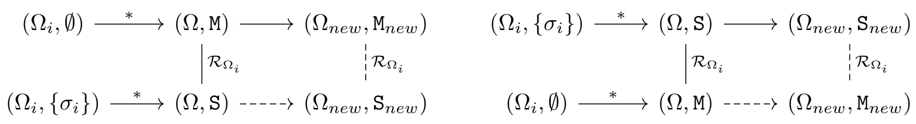

(a) Precise to History-preserving Simulation

(b) History-preserving to Precise Simulation

Fig. 4: Simulation Schema

*and consider that there exists a state* $(\Omega, \mathtt{S})$ *of the history preserving semantics of Figure 3 such that they are related by the simulation relation*

$$(\Omega, \mathtt{M})\ \mathcal{R}_{\Omega_i}\ (\Omega, \mathtt{S})$$

*We can conclude that, as illustrated in Figure 4a, there exists a state* $(\Omega_{new}, \mathtt{S}_{new})$ *such that*

$$(\Omega, \mathtt{S}) \to (\Omega_{new}, \mathtt{S}_{new}) \qquad \text{and} \qquad (\Omega_{new}, \mathtt{M}_{new})\ \mathcal{R}_{\Omega_i}\ (\Omega_{new}, \mathtt{S}_{new})$$

We will now consider the lemma showing the inverse relation. To that end we will consider a special case of the semantics of Figure 2 where instead of applying the Operation rule, we will always apply the Op & Broadcast rule, and instead of the Merge rule, we will apply Merge & Broadcast. As we mentioned before, this is equivalent to the application of the Operation/Merge rule, followed by a sequence of applications of Send. The reason we will do this is that we are interested in showing that for any execution of the semantics in Figure 3 there is an equivalent (simulated) execution of the semantics of Figure 2. Since all states can be merged in the semantics of Figure 3 we have to assume that in the semantics of Figure 2 the states have been sent with messages. Fortunately, we can choose how to instantiate the existential send messages to apply the rules as necessary, and that justifies this choice.

**Lemma 4 (Messages-semantics simulates State-semantics).** *Consider a reachable state* $(\Omega, \mathtt{S})$ *from the initial state* $\Omega_i$ *in the semantics of Figure 3. Consider moreover that according to that semantics there exists a transition of the form*

$$(\Omega, \mathtt{S}) \to (\Omega_{new}, \mathtt{S}_{new})$$

*and consider that there exists a state* $(\Omega, \mathtt{M})$ *of the state-preserving semantics of Figure 3 such that they are related by the simulates relation*

$$(\Omega, \mathtt{M})\ \mathcal{R}_{\Omega_i}\ (\Omega, \mathtt{S})$$

*We can conclude that there exists a state* $(\Omega_{new}, \mathtt{M}_{new})$ *such that*

$$(\Omega, \mathtt{M}) \to (\Omega_{new}, \mathtt{M}_{new}) \qquad \text{and} \qquad (\Omega_{new}, \mathtt{M}_{new})\ \mathcal{R}_{\Omega_i}\ (\Omega_{new}, \mathtt{S}_{new})$$

As before, an illustration of this lemma is presented in Figure 4b.

We can now conclude that the two semantics are bisimilar:

**Theorem 1 (Bisimulation).** *The semantics of Figure 2 and Figure 3 are bisimilar as established by the relation defined in Definition 1.*

The theorem above justifies carrying out our proofs with respect to the semantics of Figure 3, which has fewer rules and it better aligns with our proof methodology. This is also justifies that when reasoning semantically about state-propagating object systems we can generally ignore the effects of network delays and messages.

From the standpoint of concurrency, the system model allows the execution of asynchronous concurrent operations, where each operation is executed atomically in each replica, and the aggregation of results of different operations is performed lazily as replicas exchange their state. At this point, we assume the set of states, along with the operations and merge, forms a monotonic semi-lattice. This is a sufficient condition for Strong Eventual Consistency [3, 4, 25].

We have seen that even though we achieve convergence later, there can be instances or even long periods of time during which replicas might diverge. We need to ensure that the concurrent executions are still safe. In the next section, we discuss how to ensure safety of distributed objects built on top of the system model we described.

## 4 Proving Invariants

In this section, we report our invariant verification strategy. Specifically, we consider the problem of verifying *invariants* of highly-available distributed objects.

To support the verification of invariants we will consider a syntactic-driven approach based on program logic. Bailis et al.[2] identifies necessary and sufficient run-time conditions to establish the security of application invariants for highly-available distributed databases in a criterion dubbed $I$-confluence. Moreover, they consider the validity of a number of typical invariants and applications. Our work improves on the $I$-confluence criterion defined in [2] by providing a static, syntax-driven, and mostly-automatic mechanism to verify the correctness of an invariant for an application. We will address the specific differences in Section 7, the related work.

An important consequence of our verification strategy is that while we are proving invariants about a concurrent highly-distributed system, our verification conditions are modular (on the number of API operations), and can be carried out using standard sequential Hoare-style reasoning. These verification conditions in turn entail stability of the assertions as one would have in a logic like Rely/Guarantee.

Let us start by assuming that a given initial state for the object is denoted $\sigma_i$. Initially, all replicas have $\sigma_i$ as their local state. As explained earlier, each replica executes a sequence of state transitions, due either to a local update or to a merge incorporating remote updates.

Let us call *safe state* a replica state that satisfies the invariant. Assuming the current state is safe, any update (local or merge) must result in a safe state. To ensure this, every update is equipped with a precondition that disallows any unsafe execution.[5] Thus, a local update executes only when, at the origin replica, the current state is safe and its precondition currently holds.

Formally, an update $u$ (an operation or a merge), mutates the local state $\sigma$, to a new state $\sigma_{new} = u(\sigma)$. To preserve the invariant, `Inv`, we require that the local state respects the precondition of the update, $\mathsf{Pre}_u$: $\sigma \in \mathsf{Pre}_u \implies u(\sigma) \in Inv$

To illustrate local preconditions, consider an operation `close_auction(w: BidId)`, which sets auction status to `CLOSED` and the winner to `w` (of type BidId). The developer may have written a precondition such as `status` = `ACTIVE` because closing an auction doesn't make sense otherwise. In order to ensure the invariant that the winner has the highest amount, one needs to strengthen it with the clause `is_highest(Bids, w)`, defined as

```
∀ b ∈ Bids, b.placed ⟹ b.Amount ≤ w.Amount
```

Similarly, merge also needs to be safe. To illustrate merge precondition, let us use our running example. We wish to maintain the invariant that the highest bid is the winner. Assume a scenario where the local replica declared a winner and closed the auction. An incoming state from a remote replica contains a bid with a higher amount. When the two states are merged, we see that the resulting state is unsafe. So we must strengthen the merge operation with a precondition. The strengthened precondition looks like this:

```
status = CLOSED ⟹ ∀ Bids ∈ ℙ(Bids), is_highest(Bids, w)
∧ status′ = CLOSED ⟹ ∀ Bids ∈ ℙ(Bids), is_highest(Bids, w′)
```

This means that if the status is `CLOSED` in either of the two states, the winner should be the highest bid in any state. This condition ensures that when a winner is declared, it is the highest bid among the set of bids in any state at any replica.

Since merge can happen at any time, it must be the case that its precondition is always true, i.e., it constitutes an additional invariant. We call this as the *concurrency invariant*. Now our global invariant consists of two parts: first, the invariant ($\mathsf{Inv}$), and second, the concurrency invariant($\mathsf{Inv}_{conc}$).

### 4.1 Invariance Conditions

The verification conditions in Figure 5 ensure that for any reachable local state of a replica, the global invariant $\mathsf{Inv} \wedge \mathsf{Inv}_{conc}$, is a valid assertion. We assume the invariant to be a Hoare-logic style assertion over the state of the object. In a nutshell, all of these conditions check (i) the precondition of each of the operations, and that of the merge operation uphold the global invariant, and (ii) the global invariant of the object consists of the invariant and the concurrency invariant (precondition of `merge`).

We will develop this intuition in what follows. Let us now consider each of the rules:

[5] Technically, this is at least the weakest-precondition of the update for safety. It strengthens any *a priori* precondition that the developer may have set.

$$\sigma_i \models \mathsf{Inv} \tag{1}$$

$$\forall\ \mathsf{op}, \sigma, \sigma_{new},\ \begin{pmatrix} \sigma \models \mathsf{Pre}_{\mathsf{op}} \wedge \\ \sigma \models \mathsf{Inv} \wedge \\ [\![\mathsf{op}]\!](\sigma) = \sigma_{new} \end{pmatrix} \Rightarrow \sigma_{new} \models \mathsf{Inv} \tag{2}$$

$$\forall\ \sigma, \sigma', \sigma_{new},\ \begin{pmatrix} (\sigma, \sigma') \models \mathsf{Pre}_{\texttt{merge}} \wedge \\ \sigma \models \mathsf{Inv} \wedge \\ \sigma' \models \mathsf{Inv} \wedge \\ [\![\texttt{merge}]\!](\sigma, \sigma') = \sigma_{new} \end{pmatrix} \Rightarrow \sigma_{new} \models \mathsf{Inv} \tag{3}$$

$$(\sigma_i, \sigma_i) \models \mathsf{Inv}_{conc} \tag{4}$$

$$\forall\ \mathsf{op}, \sigma, \sigma', \sigma_{new},\ \begin{pmatrix} \sigma \models \mathsf{Pre}_{\mathsf{op}} \wedge \\ (\sigma, \sigma') \models \mathsf{Inv}_{conc} \wedge \\ [\![\mathsf{op}]\!](\sigma) = \sigma_{new} \end{pmatrix} \Rightarrow (\sigma_{new}, \sigma') \models \mathsf{Inv}_{conc} \tag{5}$$

$$\forall\ \sigma, \sigma', \sigma_{new},\ \begin{pmatrix} (\sigma, \sigma') \models \mathsf{Pre}_{\texttt{merge}} \wedge \\ (\sigma, \sigma') \models \mathsf{Inv}_{conc} \wedge \\ [\![\texttt{merge}]\!](\sigma, \sigma') = \sigma_{new} \end{pmatrix} \Rightarrow (\sigma_{new}, \sigma') \models \mathsf{Inv}_{conc} \tag{6}$$

Fig. 5: Invariant Conditions

– Clearly, the initial state of the object must satisfy the global invariant, this is checked by conditions (1) and (4).

The rest of the rules perform a kind of inductive reasoning. Assuming that we start in a state that satisfies the global invariant, we need to check that any state update preserves the validity of said invariant. Importantly, this reasoning is not circular, since the initial state is known by the rule above to be safe.[6]

– Condition (2) checks that each of the operations, when executed starting in a state satisfying its precondition and the invariant, is safe. Notice that we require that the precondition of the operation be satisfied in the starting state. This is the core of the inductive argument alluded to above, all operations – which as we mentioned in Section 3 execute atomically w.r.t. concurrency – preserve the invariant $\mathsf{Inv}$.

Other than the execution of operations, the other source of local state changes is the execution of the merge function in a replica. It is not true in general that for any two given states of an object, the merge should compute a safe state. In particular, it could be the case that the merge function needs a precondition that is stronger than the conjunction of the invariants in the two states to be merged. The following rules deal with these cases.

– We require the merge function to be annotated with a precondition strong enough to guarantee that `merge` will result in a safe state. Generally, this

[6] Indeed, the proof of soundness of program logics such as Rely/Guarantee are typically inductive arguments of this nature.

precondition can be obtained by calculating the weakest precondition [9] of `merge` w.r.t. the desired invariant. Since `merge` is the only operation that requires two states as input, the precondition of merge has two states. We can then verify that merging two states is safe. This is the purpose of rule (3).

As per the program model of Section 3, any two replicas can exchange their states at any given point of time and trigger the execution of a merge operation. Thus, it must be the case that the precondition of the merge function is enabled at all times between any two replica local states. Since merge is the only point where a local replica can observe the result of concurrent operations in other replicas, we call this a *concurrency invariant* ($\mathsf{Inv}_{conc}$). In other words: the *concurrency invariant is part of the global invariant* of the object. This is the main insight that allows us to reduce the proof of the distributed object to checking that both the invariant $\mathsf{Inv}$ and the concurrency invariant $\mathsf{Inv}_{conv}$ are global invariants. In particular, the latter implies the former, but for exposition purposes we shall preserve the invariant $\mathsf{Inv}$ in the rules.

– Just as we did with the operations above, we now need to check that whenever we have a pair of states that satisfy the concurrency invariant, if one of these states changes, the resulting pair still satisfies the concurrency invariant. This is exactly the purpose of rule (5) in the case where the state change originates from an operation execution in one of the replicas of the pair. This rule is similar to rule (2) above, where the invariant $\mathsf{Inv}$ has been replaced by $\mathsf{Inv}_{conc}$, and consequently we have a pair of states.
– Finally, as we did with rule (3), we need to check the case where one of the states of a pair of states satisfying $\mathsf{Inv}_{conc}$ is updated because of yet another merge happening (w.r.t. yet another replica) in one of these states. This is the purpose of rule (6) which is similar to rule (3), with $\mathsf{Inv}$ replaced for $\mathsf{Inv}_{conc}$.

As anticipated at the beginning of this section, the reasoning about the concurrency is performed in a completely local manner, by carefully choosing the verification conditions, and it avoids the stability blow-up commonly found in concurrent program logics. The program model, and the verification conditions allow us to effectively reduce the problem of verifying safety of an asynchronous concurrent distributed system, to the modular verification of the global invariant ($\mathsf{Inv} \wedge \mathsf{Inv}_{conc}$) as pre and post conditions of all operations and merge.

**Proposition 1 (Soundness).** *The proof rules in equations (1)-(6) guarantee that the implementation is safe.*

To conduct an inductive proof of this lemma we need to strengthen the argument to include the set of observed states as given by the semantics of Figure 3.

**Lemma 5 (Strengthening of Soundness).** *Assuming that the equations (1)-(6) hold for an implementation of a replicated object with initial state $\Omega_i$. For any state $(\Omega, \mathtt{S})$ reachable from $(\Omega_i, \{\sigma_i\})$, that is $(\Omega_i, \{\sigma_i\}) \xrightarrow{*} (\Omega, \mathtt{S})$, we have that:*

1. *for all states* $\sigma, \sigma' \in \mathtt{S}$, $(\sigma, \sigma') \models \mathsf{Inv}_{conc}$, *and*
2. *for any state* $\sigma \in \mathtt{S}$, $\sigma \models \mathsf{Inv}$.

**Corollary 1.** *The soundness proposition (1) is a direct consequence of Lemma 5.*

We remark at this point that there are numerous program logic approaches to proving invariants of shared-memory concurrent programs, with Rely/Guarantee [15] and concurrent separation logic [6] underlying many of them. While these approaches could be adapted to our use case (propagating-state distributed systems), this adaptation is not evident. As an indication of this complexity: one would have to predicate about the different states of the different replicas, restate the invariant to talk about these different versions of the state, encode the non-deterministic behaviour of merge, etc. Instead, we argue that our specialised rules are much simpler, allowing for a purely sequential and modular verification that we can mechanise and automate. This reduction in complexity is the main theoretical contribution of this paper.

### 4.2 Applying the proof rule

Let us apply the proof methodology to the auction object. Its invariant is the following conjunction:

1. Only an `ACTIVE` auction can receive bids, and
2. the highest bid, also unique, wins when the auction is `CLOSED`.

Computing the weakest precondition of each update operation, for this invariant is obvious. For instance, as discussed earlier, `close_auction(w)` gets precondition `is_highest(Bids, w)`, because of Invariant Item 2 above.

Despite local updates to each replica respecting the invariant $\mathsf{Inv}$, Figure 1 showed that it is susceptible of being violated by merging. This is the case if Bob's \$100 bid in Brussels wins, even though Charles concurrently placed a \$105 bid in Calgary; this occurred because `status` became `CLOSED` in Brussels while still `ACTIVE` in Calgary. The weakest precondition of merge for safety expresses that, if `status` in either state is `CLOSED`, the winner should be the bid with the highest amount in both the states. This merge precondition, now called the concurrency invariant, strengthens the global invariant to be safe in concurrent executions.

Let us now consider how this strengthening impacts the local update operations. Since starting the auction doesn't modify any bids, the operation trivially preserves it. Placing a bid might violate $\mathsf{Inv}_{conc}$ if the auction is concurrently closed in some other replica; conversely, closing the auction could also violate $\mathsf{Inv}_{conc}$, if a higher bid is concurrently placed in a remote replica. Thus, the auction object is safe when executed sequentially, but it is unsafe when updates are concurrent. This indicates the specification has a bug, which we now proceed to fix.

### 4.3 Concurrency Control for Invariant Preservation

As we discussed earlier, the preconditions of operations and merge are strengthened in order to be sequentially safe. An object must also preserve the concurrency invariant in order to ensure concurrent safety. Violating this indicates the presence of a concurrency bug in the specification. In that case, the operations that fail to preserve the concurrency invariant might need to synchronise. The developer adds the required concurrency control mechanisms as part of the state in our model. The modified state is now composed of the state and the concurrency control mechanism.

Recall that in the auction example, placing bids and closing the auction did not preserve the precondition of merge. This requires strengthening the specification by adding a concurrency control mechanism to restrict these operations. We can enforce them to be strictly sequential, thereby avoiding any concurrency at all. But this will affect the availability of the object.

A concurrency control can be better designed with the workload characteristics in mind. For this particular use case, we know that placing bids are much more frequent operations than closing an auction. Hence we try to formulate a concurrency control like a readers-writer lock. In order to realise this we distribute tokens to each replica. As long as a replica has the token, it can allow placing bids. Closing the auction requires recalling the tokens from all replicas. This ensures that there are no concurrent bids placed and thus a winner can be declared, respecting the invariant. The addition of this concurrency control also updates the $\mathsf{Inv}_{conc}$. Clearly, all operations must respect this modification for the specification to be considered safe.

Note that the token model described here restricts availability in order to ensure safety. Adding efficient synchronization is not a problem to be solved only with application specification in hand, it rather requires the knowledge of the application dynamics such as the workload characteristics and is part of our future work.

Figure 6 shows the evolution of the modified auction object with concurrency control. The keys shown are the tokens distributed to each replica. When a replica wants to close the auction, it can request tokens from other replicas. When a replica releases its token, it is indicated by a cross mark on the key. This concurrency control mechanism makes sure that the object is safe during concurrent executions as well. The specification including the concurrency control is given in the extended version[23].

To summarize, all updates (operations and merge) have to respect the global invariant ($\mathsf{Inv} \wedge \mathsf{Inv}_{conc}$). If an update violates $\mathsf{Inv}$, the developer must strengthen its precondition. If an update violates $\mathsf{Inv}_{conc}$, the developer must add concurrency control mechanisms.

## 5 Case Studies

This section presents three representative examples of different consistency requirements of several distributed applications. The consensus object is an ex-

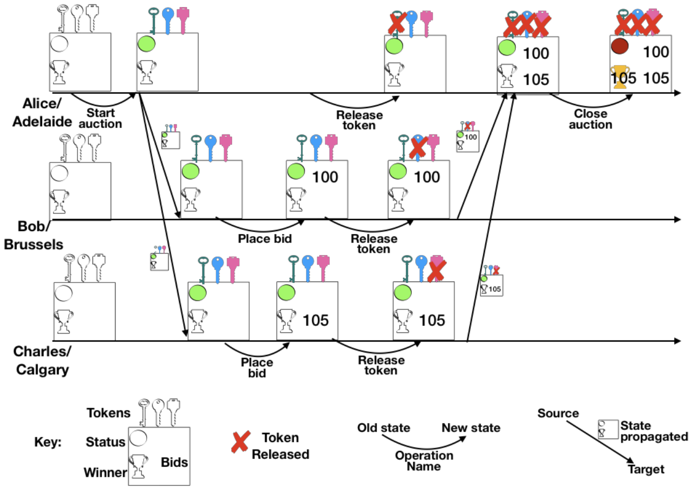

Fig. 6: Evolution of state in an auction object with concurrency control ample of a coordination-free design, illustrating a safe object with just eventual consistency. The next example of a distributed lock shows a design that maintains a total order, illustrating strong consistency. And the final example of courseware shows a mix of concurrent operations and operations with restrained concurrency. This example, similar to our auction example, illustrates applications that might require coordination for some operations to ensure safety.

For each case study, we give an overview of the operational semantics informally. We then discuss how the design preserves the safety conditions discussed in Section 4. We also provide pseudocode for better comprehension.

## 5.1 Consensus application

Consensus is required in distributed systems when all replicas have to agree upon a single value. We consider the specification of a consensus object with a fixed number of replicas. We assume that replica failures are solved locally by redundancy or other means, and all replicas participate.

The state consists of a boolean flag indicating the result of consensus, and a boolean array indicating the votes from replicas. Each replica agrees on a proposal by setting its dedicated entry in the boolean array. A replica cannot withdraw its agreement. A replica sets the consensus flag when it sees all entries of the boolean array set.

The consistency between the values of agree flag and the boolean array is ensured by the invariant. The merge function is the disjunction of the individual components. In this case study, we can see that the merge ensures safety without any additional precondition. This means that the object is trivially safe under concurrent executions.

```
Initial state:
  ¬B ∧ ¬flag

Invariant:
  flag ⟹ B

{Pre_merge: True}
# no precondition
merge(B, flag, B0, flag0):
    B := B ∨ B0
    flag := flag ∨ flag0

Comparison function:
  flag ∨ (¬flag0 ∧ (B ∨ ¬B0))

{Pre_mark: True}
# no precondition
mark():
  B.me := true

{Pre_agree: B}
agree():
  flag := true
```

Fig. 7: Pseudocode for consensus

```
Initial state:
  ∃ r, V.r ∧ t = 0

{Pre_transfer: V.me}
transfer(r_o):
  t = t+1
  V.me := false
  V.r0 := true

Comparison function:
  t > t0
  ∨ (t = t0 ∧ V = V0)

{Pre_merge :
  (t = t0 ⟹ V = V0)
  ∧ (V.me ⟹ t ≥ t0)}
merge((t,V),(t0,V0)):
  t = max(t,t0)
  v = (t0<t)?V:V0

Invariant:
  ∃ r, V.r ∧ ∀ r, r0, (V.r ∧ V.r0) ⟹ r = r0
```

Fig. 8: Specification of a distributed lock

The pseudo code of the consensus example is shown in Figure 7. The design for consensus can be relaxed, requiring only the majority of replicas to mark their boxes. The extension for that is trivial.

## 5.2 A replicated concurrency control

We now discuss an object, a distributed lock, that ensures mutual exclusion. We use an array of boolean values, one entry per replica, to model a lock. If a replica owns the lock, the corresponding array entry is set to true. The lock is transferred to any other replica by using the transfer function. The full specification is shown in Figure 8.

We need to ensure that the lock is owned by exactly one replica at any given point in time, which is the invariant here. For simplicity, we are not considering failures. In order to preserve safety, we need to enforce a precondition on the transfer operation such that the operation can only transfer the ownership of its origin replica. For state inflation, a timestamp associated with the lock is incremented during each transfer.

A merge of two states of this distributed lock will preserve the state with the highest timestamp. In order for the merge function to be the least upper bound, we must specify that if the timestamps of the two states are equal, their corresponding boolean arrays are also equal. Also if the origin replica owns the lock, it has the highest timestamp. The conjunction of these two restrictions which form the precondition of merge, $\mathsf{Pre}_{\texttt{merge}}$, is the concurrency invariant, $\mathsf{Inv}_{conc}$.

Consider the case of three replicas $r_1$, $r_2$ and $r_3$ sharing a distributed lock. Assume that initially replica $r_1$ owns the lock. Replicas $r_2$ and $r_3$ concurrently place a request for the lock. The current owner $r_1$, has to make a decision on the priority of the requests based on the business logic. $r_1$ calculates a higher priority for $r_3$ and transfers the lock to $r_3$. Since $r_1$ no longer has the lock, it cannot issue any further transfer operations. We see here clearly that the transfer operation is safe. In the new state, $r_3$ is the only replica that can perform a transfer operation. We can also note that this prevents any concurrent transfer operations. This can guarantee mutual exclusion and hence ensures safety in a concurrent execution environment.

An interesting property we can observe from this example is total order. Due to the preconditions imposed in order to be safe, we see that the states progress through a total order, ordered by the timestamp. The transfer function increases the timestamp and merge function preserves the highest timestamp.

### 5.3 Courseware

We now look at an application that allows students to register and enroll in a course. For space reasons, we elide the pseudocode which can be found in the extended version[23]. The state consists of a set of students, a set of courses and enrollments of students for different courses. Students can register and deregister, courses can be created and deleted, and a student can enroll for a course. The invariant requires enrolled students and courses to be registered and created respectively.

The set of students and courses consists of two sets - one to track registrations or creations and another to track deregistrations or deletions. Registration or creation monotonically adds the student or course to the registered sets respectively and deregistration or deletion monotonically adds them to the unregistered sets. The semantics currently doesn't support re-registration, but that can be fixed by using a slightly modified data structure that counts the number of times the student has been registered/unregistered and decides on the status of registration. Enrollment adds the student-course pair to the set. Currently, we do not consider canceling an enrollment, but it is a trivial extension. Merging two states takes the union of the sets.

Let us consider the safety of each operation. The operations to register a student and create a course are safe without any restrictions. Therefore they do not need any precondition. The remaining three operations might violate the invariant in some cases. This leads to strengthening their preconditions. The precondition of the operation for deregistering a student and deleting a course requires no existing enrollments for them. For enrollment, both the student and the course should be registered/created and not unregistered/deleted.

Merge also requires strengthening of its precondition. It requires the set of enrolled students and courses to be registered and not unregistered in all the remote states as well. This is the concurrent invariant ($\mathsf{Inv}_{conc}$) for this object.

Running this specification through our tool which we describe in Section 6 reveals concurrency issues for deregistering a student, deleting a course and enrollment. This means that we need to add concurrency control to the state.

For this use case, we know that enrolling will be more frequent than deregistering a student or deleting a course. So, we model a concurrency control mechanism as in the case of the auction object discussed earlier. We assign a token to each replica for each student and course, called a student token and course token respectively. A replica will have a set of student tokens indicating the registered students and course tokens indicating the created courses. In order to deregister a student or delete a course, all replicas must have released their tokens for that particular student/course. Enroll operations can progress as long as the student token and course token are available at the local replica for the student and course for that particular enrollment.

This concurrency control mechanism now forms part of the state. The preconditions of operations and merge are recomputed and the concurrency invariant is updated. The edited specification passes all checks and is deemed safe.

## 6 Automation

In this section, we present a tool to automate the verification of invariants as discussed in the previous sections. Our tool, called *Soteria* is based on the Boogie [5] verification framework. The input to Soteria is a specification of the object written as Boogie procedures, augmented with a number of domain-specific annotations needed to check the properties described in Section 4.

Let us now consider how a distributed object is specified in Soteria.:

- **State:** We require the programmer to provide a declaration of the state using the global variables in Boogie. The data types can be either built-in or user defined.
- **Comparison function:** Next we require the programmer to provide a comparison function. This function determines the partial order on states. Again, we shall use this comparison function as a basis to check the lattice conditions, and whether each operation is an inflation on the lattice. We use the keyword `@gteq` to annotate the comparison function in the tool. This comparison function returns true when all the components of the first state are greater than or equal to the corresponding components in the other state. It is encoded as a function in Boogie.
- **Operations:** We require the programmer to provide the implementation of the operations of the object. Moreover, for each operation `op` we require the programmer to provide the precondition $\mathsf{Pre}_{\mathsf{op}}$. In general, operations are encoded as Boogie procedures. Alternatively, we could just require only a post-condition describing how the state transitions from the precondition to the post-condition. Notice that since in our program model operations are atomic, this is an unambiguous encoding of the operations.
A few things are important in this code. The specification declares operations that can modify the contents of the global variables as declared in the `modifies` clause. Preconditions are annotated with the `requires` clauses, and the postcondition is specified by the `ensures` clauses. The semantics of multiple `requires` and `ensures` clauses is conjunction.
- **Merge function:** We require the special `merge` operation to be distinguished from other operations. To that end, we use the annotation `@merge`. While, as mentioned before, the precondition of `merge` can be obtained by calculating the weakest precondition to ensure safety. The current version of Soteria does not perform this step automatically, it relies on the developer to provide the preconditions. Notice that, as we argued in Section 4.1, Soteria will consider this as the concurrency invariant ($\mathsf{Inv}_{conc}$).
While in Section 3 we mentioned that the `merge` procedure takes two states as arguments, in the specification input to Soteria, the procedure merge takes only one state as the argument. This is because this procedure assumes that the merge is being applied in a replica, and therefore, the local state of the replica is captured by the global variables.
- **Invariant:** Clearly, we require the programmer to provide the invariant to be verified by the tool. This invariant is simply provided as a Boogie assertion over the state of the object. Once more, we require the invariant to be annotated with the special keyword `@invariant`.

While these are the components required by Soteria to check the safety, often Boogie requires additional information to verify the procedures. Some of these components are:

- User-defined data types,
- Constants to declare special objects such as the origin replica me, or to bound the quantifiers,
- We sometimes make recourse to inductively-defined functions over aggregate data structures, for instance, to obtain the maximum in a set of values. Since we would like to use these functions in the specifications, we axiomatise their semantics to enable the SMT solver used by Boogie to discharge our proof obligations. This is particularly important for list comprehensions, and array operations. We follow the approach of Leino et al.[18].
- When we iterate over lists, arrays or matrices, we need to provide Boogie with loop invariants. Loops are part of the programs, and thus, verified by Boogie.

### 6.1 Verification passes

The verification of a specification is performed in multiple stages. Let us consider these in order:

1. **Syntax checks**
   The first simple checks validate that the specification provided respects Boogie syntax when ignoring Soteria annotations. It also calls Boogie to validate that the types are correct and that the pre/post conditions provided are sound.
   Then it checks that the specification provides all the elements necessary for a complete specification. Specifically, it checks the function signatures marked by `@gteq` and `@invariant` and the procedure marked by `@merge`.
2. **Convergence check**
   This stage checks the convergence of the specification. Specifically, it checks whether the specification respects Strong Eventual Consistency. The *Strong Eventual Consistency* (SEC) property states that any two replicas that received the same set of updates are in the same state. To guarantee this, objects are designed to have certain sufficient properties in the encoding of the state [3, 4, 25], which can be summarised as follows:
   - The state space is equipped with an ordering operator, comparing two states.
   - The ordering forms a join-semilattice.
   - Each individual operation is an inflation in the semilattice.
   - The `merge` operation, composing states from two replicas, computes the least-upper-bound of the given states in the semilattice.

We present the conditions formally in the extended version[23].
   An alternative is to make use of the CALM theorem [12]. This allows non-monotonic operations, but requires them to coordinate. However, our aim is to provide maximum possible availability with SEC. [7]
   To ensure these conditions of Strong Eventual Consistency, the tool performs the following checks:
   - That each operation is an inflation. In a nutshell, we prove using Boogie the following Hoare-logic triple:

$\texttt{assume}\ \ \sigma \in \mathsf{Pre_{op}}$
     $\texttt{call}\ \ \sigma_{new} := \mathsf{op}(\sigma)$
     $\texttt{assert}\ \ \sigma_{new} \geq \sigma$

- Merge computes the least upper bound. The verification condition discharged is shown below:

$\texttt{assume}\ \ (\sigma, \sigma') \in \mathsf{Pre_{merge}}$
     $\texttt{call}\ \ \sigma_{new} := \mathtt{merge}(\sigma, \sigma')$
     $\texttt{assert}\ \ \sigma_{new} \geq \sigma \wedge \sigma_{new} \geq \sigma'$
     $\texttt{assert}\ \ \forall \sigma*, \sigma* \geq \sigma \wedge \sigma* \geq \sigma' \implies \sigma* \geq \sigma_{new}$

3. **Safety check** This stage verifies the safety of the specification as discussed in Section 4. This stage is divided further into two sub-stages:
   - *Sequential safety:* Soteria checks whether each individual operation is safe. This corresponds to the conditions (2) and (3) in Figure 5. The verification condition discharged by the tool to ensure sequential safety of operations is:

[7] Convergence of our running example is discussed in the extended version[23].

$$\begin{array}{l} \texttt{assume}\ \ \sigma \in \mathsf{Pre}_{\mathsf{op}} \wedge \mathsf{Inv} \\ \texttt{call}\ \ \sigma_{new} := \mathsf{op}(\sigma) \\ \texttt{assert}\ \ \sigma_{new} \in \mathsf{Inv} \end{array}$$

The special case of the `merge` function is verified with the following verification condition:

$$\begin{array}{l} \texttt{assume}\ \ (\sigma, \sigma') \in \mathsf{Pre}_{\texttt{merge}} \wedge \sigma \in \mathsf{Inv} \wedge \sigma' \in \mathsf{Inv} \\ \texttt{call}\ \ \sigma_{new} := \texttt{merge}(\sigma, \sigma') \\ \texttt{assert}\ \ \sigma_{new} \in \mathsf{Inv} \end{array}$$

Notice that in this condition we assume that there are two copies of the state, the state of the replica applying the merge, and the state with superscript representing a state arriving from another replica. In case of failure of the sequential safety check, the designer needs to strengthen the precondition of the operation (or merge) which was unsafe.

– *Concurrent safety:* Here we check whether each operation upholds the precondition of merge. This corresponds to the conditions (5) and (6) in Figure 5. Notice that while this check relates to the concurrent behaviour of the distributed object, the check itself is completely sequential; it does not require reasoning about operations performed by other processes. As shown in Section 4, this ensures safety during concurrent operation. The verification conditions are:

$$\begin{array}{l} \texttt{assume}\ \ \sigma \in \mathsf{Pre}_{\mathsf{op}} \wedge \mathsf{Inv} \wedge (\sigma, \sigma') \in \mathsf{Inv}_{conc} \\ \texttt{call}\ \ \sigma_{new} := \mathsf{op}(\sigma) \\ \texttt{assert}\ \ (\sigma_{new}, \sigma') \in \mathsf{Inv}_{conc} \end{array}$$

to validate each operation `op`, and

$$\begin{array}{l} \texttt{assume}\ \ (\sigma, \sigma') \in \mathsf{Inv}_{conc} \wedge \sigma \in \mathsf{Inv} \wedge \sigma' \in \mathsf{Inv} \\ \texttt{call}\ \ \sigma_{new} := \texttt{merge}(\sigma, \sigma') \\ \texttt{assert}\ \ (\sigma_{new}, \sigma) \in \mathsf{Inv}_{conc} \end{array}$$

to validate a call to `merge`. If the concurrent safety check fails, the design of the distributed object needs a replicated concurrency control mechanism embedded as part of the state.

When all checks are validated, the tool reports that the specification is safe. Whenever a check fails, Soteria provides a counterexample [8] along with the failure message tailored to the type of check. This can help the developer identify issues with the specification and fix it.

Once the invariants and specification of an application is given, Soteria is fully automatic, thanks to Z3, an SMT solver that is fully automated. The specification of the application includes the state, all the operations including the pre and post conditions (including merge). In case the invariant cannot be proven, Soteria provides counter-examples. The programmer can leverage these to update the specification with appropriate concurrency control, rerun Soteria, and so on until the application is correct. As far as the proof system is concerned, no programmer involvement is required. Currently, the effort of adding the required synchronization conditions is manual, but as the next step, we are working on

[8] Soteria uses the counter model provided by Boogie.

automating the efficient generation of synchronization control considering the workload characteristics. The tool and the full specifications in the form of the tool input are available at Soteria [22]. [9]

## 7 Related Work

Several works have concentrated on the formalisation and specification of eventually consistent systems [7, 8, 27] to mention but a few.

A number of works concentrate on the specification and correct implementation of replicated data types [10, 14]. Unlike these works, we are not concerned with the correctness of the data type implementation with respect to a specification, but rather on proving properties that hold of a distributed object.

Gotsman et al.[11] present a proof methodology for proving invariants of distributed objects. In fact, that work has been extended with a tool called CISE [24] which, similar to Soteria, performs the check using an SMT solver as a backend. Another more user-friendly tool was developed by Marcelino et al.[19] based on the principle of CISE. It is named Correct Eventual Consistency(CEC) Tool. The tool is based on Boogie verification framework and also proposes sets of tokens that the developer might use. An improved token generation by using the counterexamples generated by Boogie is discussed by Nair and Shapiro[20].

Unlike our work, CISE and CEC (and more generally the work of Gotsman et al.[11]) consider the implementation of operation-based objects. As a consequence, they assume that the underlying network model ensures causal consistency, and the proof methodology therein presented requires reasoning about concurrent behaviours (reflected as stability verification conditions on assertions). We position Soteria as a *complementary* tool to CISE, since CISE is not well-adapted to reason about systems that propagate state, and Soteria is not well-adapted to reason about objects that propagate operations. We consider, as part of our future work, the use of both CISE and Soteria in tandem to prove properties depending on the implementation of the objects at hand.

Houshmand et al.[13] extends CISE by lowering the causal consistency requirements and generating concurrency control protocols. It still requires reasoning about concurrent behaviours.

As anticipated in Section 4, Bailis et al. [2] introduced the concept of $I$-confluence based on a similar system model. $I$-confluence states that for an invariant to hold in a lattice-based state-propagating distributed application, the set of *reachable* valid (i.e. invariant preserving) states must be closed under operations and merge. This condition is similar to the ones presented in Figure 5. However, there is a fundamental difference: while Bailis et al. [2] recognises that one needs to consider only *reachable* states when checking that the merge operation satisfies the invariant, they do not provide means to identify these reachable states. This is indeed a hard problem. In Soteria, we instead *over-approximate the set of reachable states* by ignoring whether the states are indeed reachable,

[9] Experimental results with verification time is provided in the extended version[23].

but requiring that their merge satisfies the invariant. This is captured in the concurrency invariant, $\mathsf{Inv}_{conc}$, which is synthesised from the user provided invariant. How to obtain this invariant is understandably not addressed in Bailis et al.[2] since no proof technique is provided. Notice that this is a sound approximation since it guarantees the invariant is satisfied, and we also verify that every operation preserves this condition as shown in Corollary 1. In this sense we say that the pre-condition of merge for a given invariant I, is also an invariant of the system. It is this abstraction step that makes the analysis performed by Soteria to be syntax-driven, automated, and machine-checked. The fact that Soteria is an analysis of a program is in contrast with I-confluence [2] where no means to link a given program text to the semantical model, let alone rules to show that the syntax implies invariant preservation, are provided. In other words, I-confluence [2] does not provide a program logic, but rather a meta-theoretical proof about lattice-based state-propagating systems.

Our previous work [21], provides an informal proof methodology for ensuring safety of Convergent Replicated Data Types(CvRDTs), which are a group of specialised data structures used to ensure convergence in distributed programming. This work builds upon it, and formalises the proof rules and prove them sound. We relax the requirement of CvRDTs by allowing the usage of any data types, that together respect the lattice conditions mentioned in Section 3. We also show several case studies which demonstrate the use of the rule.

A final interesting remark is that we can show how our methodology can aid in the verification of distributed objects mediated by concurrency control. Some works [16, 17, 26, 27] have considered this problem from the standpoint of synthesis, or from the point of view of which mechanisms can be used to check a certain property of the system.

## 8 Conclusion

We have presented a sound proof rule to verify invariants of state-based distributed objects, i.e., the objects that propagate state. We present the proof obligations guaranteeing that the implementation is safe in concurrent execution by reducing the problem to checking that each operation of the object satisfies a precondition of the `merge` function of the state.

We presented Soteria, a tool sitting on top of the Boogie verification framework. This tool can be used to identify the concurrency bugs in the design of a distributed object. Soteria also checks convergence by checking the lattice conditions on the state, described by [3]. We have shown multiple compelling case-studies showing how Soteria can be leveraged to ensure the correctness of distributed objects that propagate state. It would be an interesting next step to look into automatic concurrency control synthesis. The synthesised concurrency control can be analysed and adapted dynamically to minimise the cost of synchronisation.

**Acknowledgements.** This research is supported in part by the RainbowFS project (*Agence Nationale de la Recherche*, France, number ANR-16-CE25-0013-01) and by European H2020 project 732 505 LightKone (2017–2020).
